# Public Health Surveillance of Pediatric Polio in Pakistan: A Cohort Study

**DOI:** 10.7759/cureus.65356

**Published:** 2024-07-25

**Authors:** Laraib Shabbir Rajput, Sana Noor, Muhammad Muneeb Khan, Mohammad Sajjad, Sidra Farooq, Ayat Ullah

**Affiliations:** 1 Department of Medicine, Dow University Hospital, Karachi, PAK; 2 Department of Community Medicine, Avicenna Medical and Dental College and Hospital, Lahore, PAK; 3 Medicine, Ayub Medical College, Abbottabad, PAK; 4 Department of Community Medicine, Gajju Khan Medical College, Shamansoor Medical Hospital, Swabi, PAK; 5 Department of Community Medicine, HITEC Institute of Medical Sciences, Taxila, PAK; 6 General Practice, Lady Reading Hospital, Peshawar, PAK

**Keywords:** paralysis, pakistan, public health surveillance, polio, pediatric

## Abstract

Background and objective

Polio continues to be endemic in Pakistan despite substantial international efforts to combat it, which presents a serious public health concern. Strategies for eradicating polio depend on understanding the dynamics of pediatric polio transmission and the efficacy of surveillance. This research study aimed to critically evaluate the public health surveillance system for pediatric polio in Pakistan and propose recommendations for improvement.

Methodology

This study was conducted from June 2020 to July 2023 in three well-known hospitals in different areas of Pakistan and involved 26 patients. Reviews of medical records, interviews, and surveillance report analysis were all part of the data collection process. Descriptive statistics, chi-square tests, and logistic regression analysis were performed using SPSS Statistics version 27.0 (IBM Corp., Armonk, NY) with the statistical significance set at p<0.05.

Results

The highest incidence of polio was observed in children aged 13-24 months (nine patients, 34.62%), with males accounting for 14 cases (53.85%) and urban residents 16 cases (61.54%). Vaccination status significantly influences disease incidence (p<0.001), with two patients (7.69%) unvaccinated, 10 patients (38.46%) partially vaccinated, and 14 patients (53.85%) fully vaccinated. Paralysis was the predominant symptom in 16 patients (61.54%). Recovery outcomes varied, with eight patients (31%) fully recovering, 12 patients (46%) showing partial improvement, and six patients (23%) experiencing chronic motor impairments. Effective surveillance depends on timely reporting [odds ratio (OR): 2.15, p<0.001] and healthcare worker training (OR: 1.67, p<0.001), highlighting crucial aspects of polio management strategies.

Conclusions

Based on our findings, vaccination status significantly impacts polio occurrence, with a notable proportion found in partially vaccinated or unvaccinated children. Paralysis remains the primary symptom, with varied recovery outcomes, including chronic motor impairments in some cases. This study underscores Pakistan's ongoing challenges with pediatric polio, highlighting the crucial need for improved vaccination, surveillance, and rehabilitation efforts.

## Introduction

Polio is an extremely contagious viral illness that primarily affects young children, especially those aged 13-24 months, and can lead to paralysis and, in severe cases, even death [1]. The disease is still widespread in Pakistan and Afghanistan despite international attempts to eliminate it [2]. Given Pakistan's significant public health efforts and foreign backing, the country's continued polio prevalence is especially concerning [3]. Devising efforts to eradicate the illness requires understanding the dynamics of polio transmission and the efficacy of public health monitoring in the area [4]. Pakistan's public health monitoring system has proven crucial in identifying and controlling polio epidemics [5]. This approach relies on community-based monitoring, reporting from health institutions, environmental surveillance via sewage samples, and both passive and active surveillance techniques [6]. International institutions like the WHO and UNICEF, which provide financial support, technical help, and immunization programs, have aided these efforts [7]. The fact that polio is still spreading despite these efforts indicates possible shortcomings and inadequacies in the monitoring system [8].

Pakistan faces several obstacles in its efforts to eradicate polio [9]. Vaccination attempts are hampered by socioeconomic challenges such as poverty, illiteracy, and lack of access to healthcare [10]. Furthermore, political and religious aspects often compound the effects of vaccine reluctance and disinformation, which result in lost immunization chances. Also, the administration of vaccinations and the gathering of monitoring data are made more difficult in certain areas by security concerns [11]. Enhancing the efficacy of polio monitoring and eradication efforts requires a thorough understanding of these intricate aspects and dynamics [12].

Prior research has concentrated on several facets of Pakistan's polio eradication efforts, such as vaccination rates, the effects of booster shots, and the function of foreign assistance [13]. Nonetheless, a thorough assessment of the public health monitoring system focusing on pediatric polio is required. An assessment of this kind might help specifically point to important deficiencies and suggest improvements to strengthen the detection and reaction systems. This research study aimed to critically evaluate the public health surveillance system for pediatric polio in Pakistan and propose recommendations for improvement.

## Materials and methods

Study design and setting

The research was conducted over three years, from July 2020 to June 2023, at three prominent hospitals across Pakistan: Pakistan Institute of Medical Sciences (PIMS) in Islamabad, Dow University Hospital in Karachi, and Lady Reading Hospital in Peshawar. These hospitals were selected due to their large catchment areas and comprehensive medical services. A total of 26 pediatric patients were included in the study. PIMS is renowned for its extensive tertiary care facilities, Dow University Hospital is a leading healthcare provider in Karachi, and Lady Reading Hospital is the largest hospital in Khyber Pakhtunkhwa, ensuring a diverse participant pool.

Inclusion and exclusion criteria

The study included all pediatric patients (age range: six months to six years) receiving treatment for poliomyelitis throughout the research period. This also included children identified through community-based and environmental monitoring as well as those identified via the polio surveillance network. Patients without complete medical records, those unable to participate in follow-up appointments, and children whose guardians or parents objected to participation were excluded. The comprehensive inclusion criteria ensured a representative sample of pediatric polio patients, while the exclusion criteria were set to maintain the integrity and reliability of the collected data.

Sample size

The sample size was determined by taking into account the frequency of pediatric polio cases and the surveillance system's capabilities. A total of 26 pediatric patients fulfilled the inclusion criteria and were included in the study. The small sample size reflects the relative rarity of poliomyelitis thanks to successful vaccination campaigns but provides critical insights into current cases and the effectiveness of ongoing surveillance and treatment strategies.

Data collection

Data collection involved a thorough review of medical records, interviews with healthcare providers, and analysis of surveillance reports. Demographic information, medical history, immunization history, clinical presentation, laboratory test results, and follow-up information were meticulously gathered. Additional data were obtained from reports by healthcare institutions, community health workers, and environmental monitoring conducted by the surveillance network. This multi-source approach ensured comprehensive data collection and enabled cross-verification of information.

Statistical analysis

SPSS Statistics version 27.0 (IBM Corp., Armonk, NY) was used for data analysis. Demographic and clinical features were summed up together by using descriptive statistics. Chi-square tests were used to compare categorical data, and logistic regression analysis was employed to identify parameters linked to the effective identification and management of polio patients. A p-value less than 0.05 was the threshold for statistical significance.

Ethical approval

The PIMS Institutional Review Board granted the research ethical clearance. All participating children's parents or guardians provided their informed consent.

## Results

The median age of the cohort was 19.5 months (IQR: 10-36; range: 6-72 months), with children between the ages of 13 and 24 months accounting for 34.62% of cases, followed by those between 6 and 12 months (26.92%) and 25 and 36 months (19.23%). Regarding gender distribution, there were more males (53.85%) than females (46.15%). Geographically, metropolitan regions accounted for the bulk of instances (61.54%) as opposed to rural areas (38.46%) (Table 1).

**Table 1:** Demographic characteristics of pediatric polio patients IQR: interquartile range

Variable		Number (N = 26)	Percentage (%)
Age group, months	6-12	7	26.92
	13-24	9	34.62
	25-36	5	19.23
	37-48	3	11.54
	49-60	1	3.85
	61-72	1	3.85
Age, months, median (IQR)	19.5 (10-36)	-	-
Age range, months	6-72	-	-
Gender	Male	14	53.85
	Female	12	46.15
Residence	Urban	16	61.54
	Rural	10	38.46

**Figure 1:**
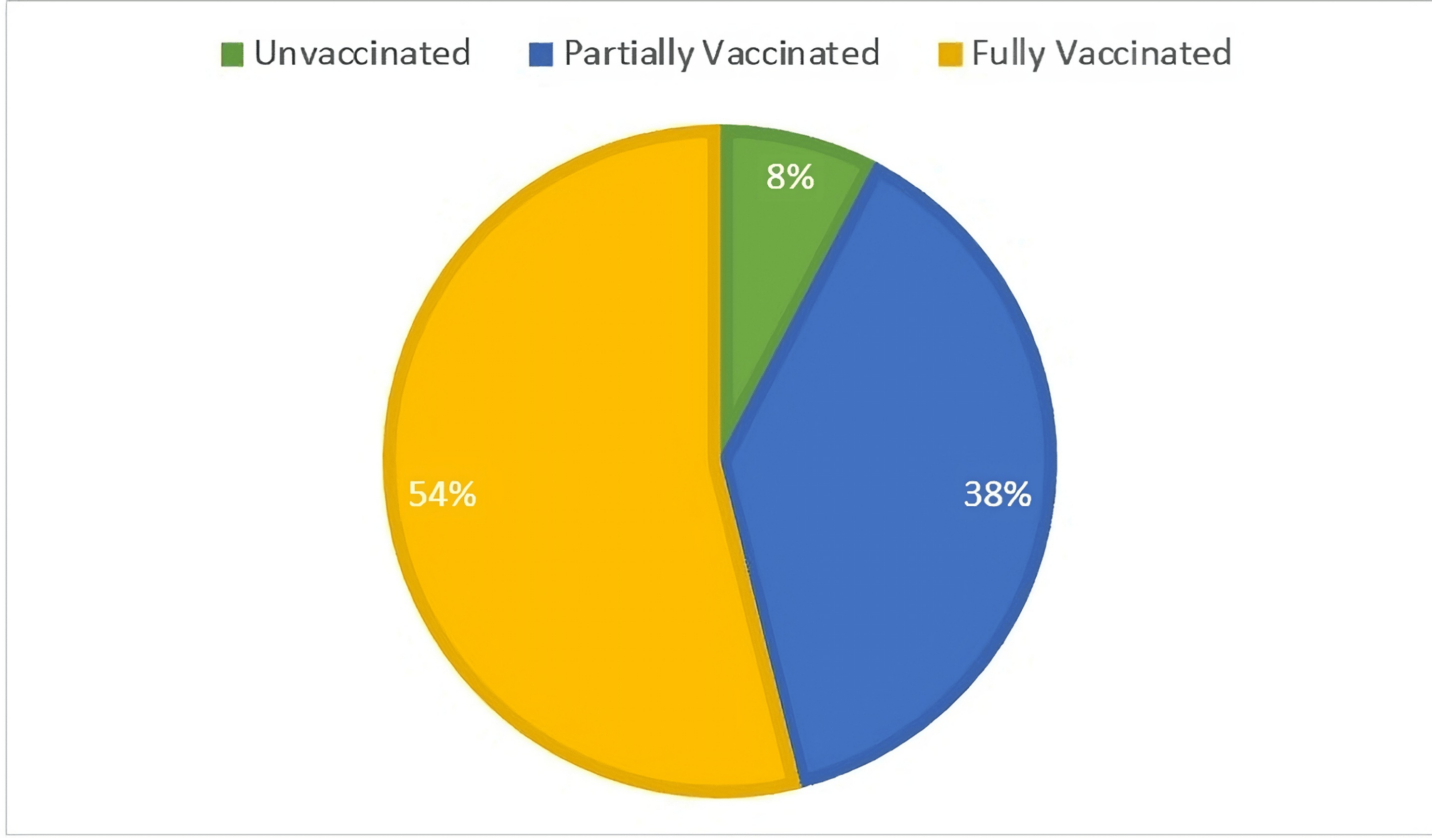
Vaccination status distribution among pediatric polio patients

The distribution of polio vaccination statuses among the 26 pediatric patients in the research was as follows: two patients (8%) had not had any polio vaccines, 10 patients (38%) had only received partial vaccinations, and 14 patients (54%) had received all recommended doses (Figure 1).

**Figure 2:**
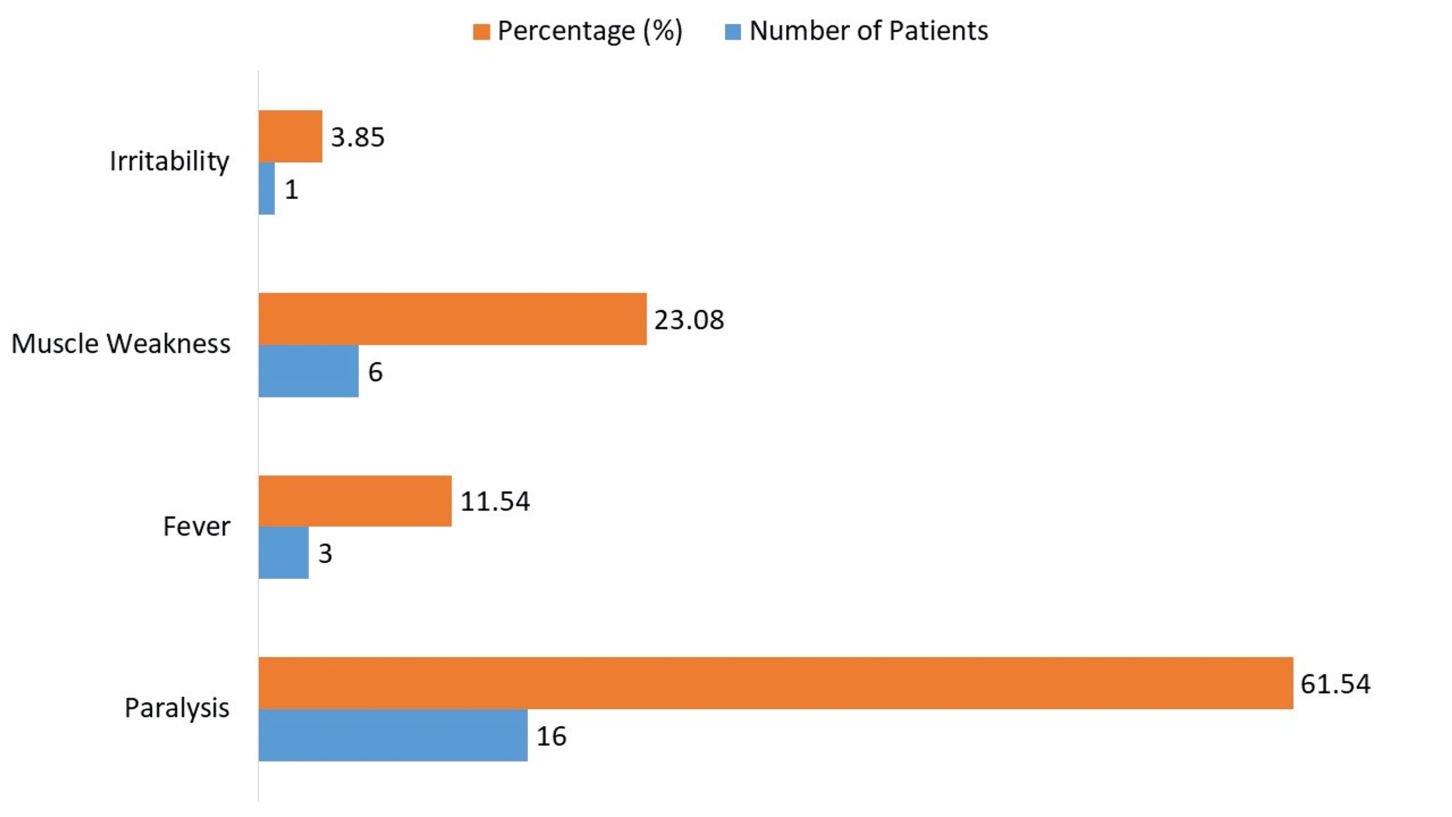
Clinical symptoms and presentations in pediatric polio cases

The clinical signs of the 26 pediatric patients varied; the most common symptom, observed in 16 individuals (61.54%), was paralysis (Figure 2). Three patients (11.54%) had a fever, while six patients (23.08%) reported having muscular weakness. In addition, one patient (3.85%) was reported to have irritation.

**Figure 3:**
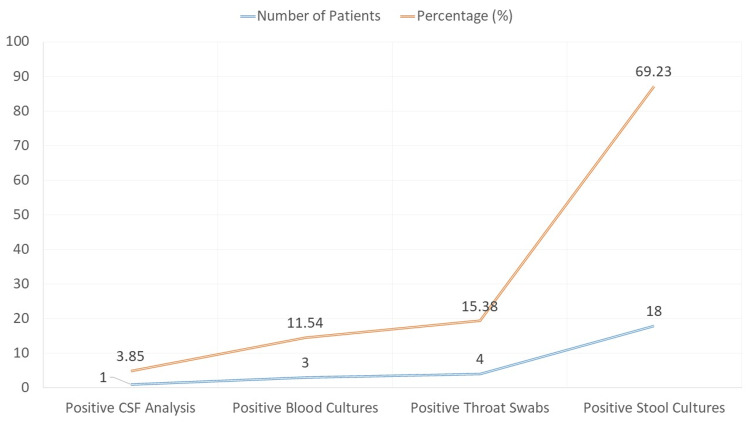
Laboratory confirmation of polio cases by test type

The findings of the laboratory tests showed variable proportions of positive results (Figure 3). The results of the examination of the patients' cerebrospinal fluid (CSF) were positive in one patient (3.85%); three patients (11.54%) had positive blood cultures, four patients (15.38%) had positive throat swabs, and 18 patients (69.23%) had positive stool cultures.

**Figure 4:**
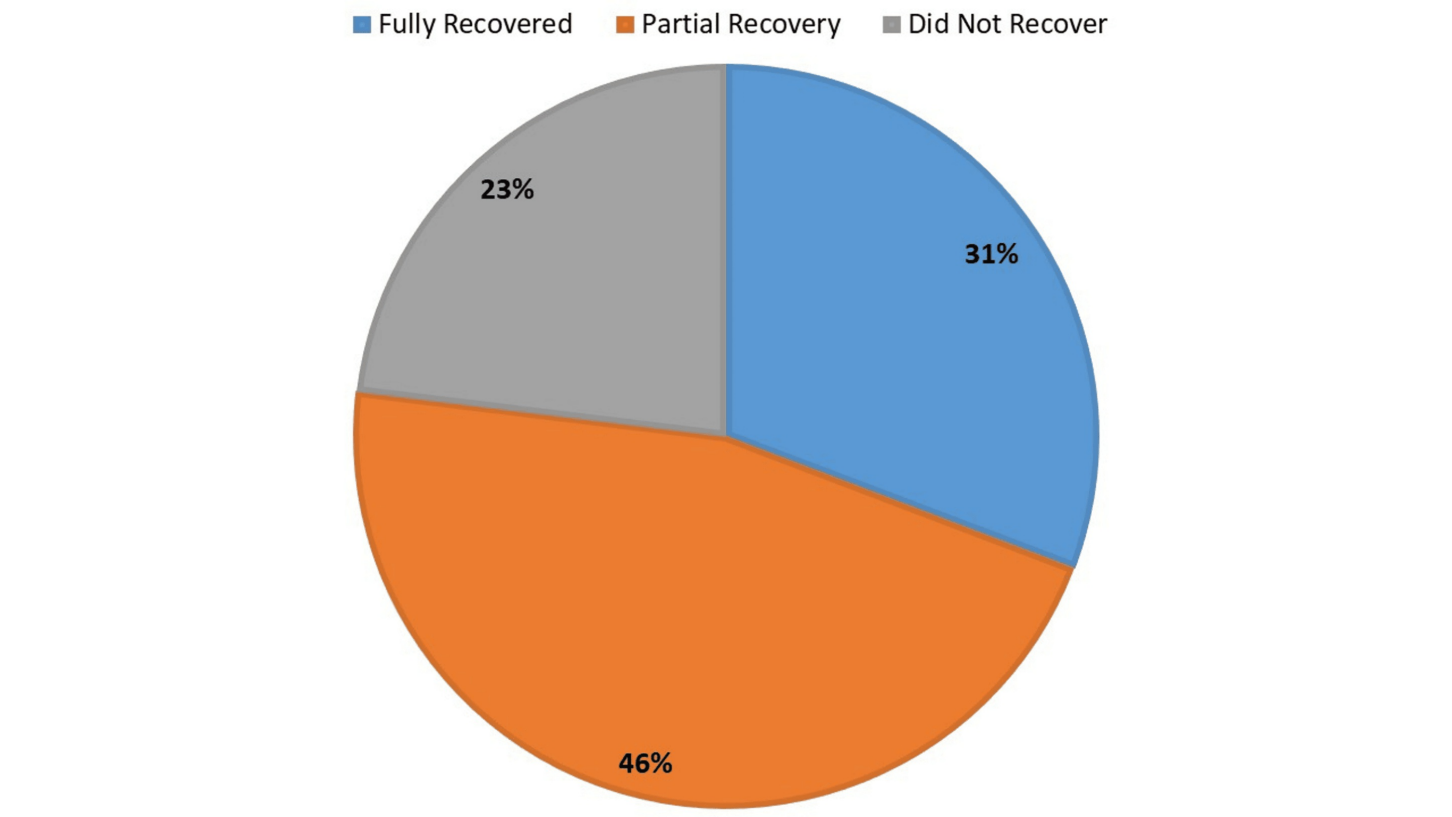
Recovery outcomes in pediatric polio patients at follow-up

Results of the follow-up of the children with polio varied: eight patients (31%) recovered completely, regaining all motor function and mobility. Furthermore, 12 patients (46%) had better symptoms but still only partial healing. Six patients (23%), on the other hand, did not fully recover, suggesting that poliomyelitis is still causing chronic motor impairments (Figure 4).

Table 2 presents the characteristics among the sample that affect detection and response in pediatric polio surveillance; 21 individuals (80.77%) had timely reporting [odds ratio (OR): 2.15, 95% confidence interval (CI): 1.78-2.59, p<0.001] and showed a substantial influence on the efficacy of surveillance. Eighteen patients (69.23%) had adequate follow-up (OR: 1.98, 95% CI: 1.65-2.37, p<0.001), highlighting the importance of this kind of follow-up in improving patient monitoring. The favorable correlation between surveillance outcomes and community engagement was highlighted by the OR of 1.45 (95% CI: 1.19-1.77, p = 0.001), which was seen in 13 patients (50.00%). An OR of 1.67 (95% CI: 1.38-2.03, p<0.001) for health worker training, which was seen in 17 cases (65.38%), demonstrated its substantial contribution to efficient polio surveillance procedures.

**Table 2:** Factors influencing detection and response in pediatric polio surveillance with logistic regression analysis CI: confidence interval

Factor	Number of patients (N = 26)	Percentage (%)	Odds ratio	95% CI	P-value
Timely reporting	21	80.77	2.15	1.78-2.59	<0.001
Adequate follow-up	18	69.23	1.98	1.65-2.37	<0.001
Community involvement	13	50.00	1.45	1.19-1.77	0.001
Health worker training	17	65.38	1.67	1.38-2.03	<0.001

The findings of the chi-square test for the categorical variables in the pediatric polio surveillance are shown in Table 3. Gender and polio cases were significantly correlated, according to the analysis (χ² = 4.23, p = 0.039), with a specific gender showing a greater frequency than the other. Additionally, there was a significant association between vaccination status and polio incidence (χ² = 16.57, p<0.001), highlighting the role that immunization plays in disease prevention. Moreover, there was a significant correlation between the place of residency and the incidence of polio (χ² = 5.89, p = 0.015), indicating that there are different risk factors in urban and rural regions. The significance of clinical presentation in polio diagnosis was noteworthy (χ² = 22.06, p<0.001), highlighting the variety of symptoms linked to the illness and its influence on response and surveillance tactics.

**Table 3:** Chi-square test results for factors associated with pediatric polio incidence

Categorical variable	Chi-square value	P-value
Gender	4.23	0.039
Vaccination status	16.57	<0.001
Residence	5.89	0.015
Clinical presentation	22.06	<0.001

## Discussion

The findings of this study underscore the persistent challenges posed by pediatric polio in Pakistan, particularly among children aged 13-24 months, who accounted for 34.62% of cases. This alignment in terms of demographics with prior research indicates a consistent vulnerability in this age group, as highlighted by Sheikh et al. [14] and Rehman et al. [15], who noted similar trends in polio susceptibility due to incomplete vaccination coverage. The fact that urban areas accounted for 61.54% of cases suggests that urban vaccination strategies may need refinement, addressing barriers to access and awareness that have been previously identified [16].

Vaccination status emerged as a significant predictor of disease incidence, with 8% of patients found to be unvaccinated, 38% partially vaccinated, and 54% fully vaccinated. This distribution not only highlights the importance of comprehensive immunization efforts but also corroborates findings from Mangal et al. [17], which emphasize that partially vaccinated populations are at a heightened risk for polio outbreaks. The statistical significance of the association between vaccination status and polio incidence (χ² = 16.57, p<0.001) reinforces the need for sustained immunization campaigns to ensure complete coverage.

Clinical manifestations in this cohort revealed paralysis as the predominant symptom, affecting 61.54% of patients, a result that aligns with previous studies identifying paralysis as a hallmark of poliovirus infection [18,19]. This symptomatology emphasizes the necessity for early detection and timely intervention, particularly in endemic regions. The significant correlation found between timely reporting and effective surveillance (OR: 2.15, 95% CI: 1.78-2.59, p<0.001) suggests that improving reporting mechanisms could substantially enhance health system responsiveness, echoing calls from Duintjer et al. [20] and Ochiai et al. [21] for improved surveillance frameworks.

Recovery outcomes varied considerably, with 31% of patients achieving full recovery while 23% experienced chronic motor impairments. This variability is consistent with other research that highlights the long-term sequelae associated with poliovirus infections, necessitating ongoing rehabilitation support for affected individuals [20-22]. Furthermore, the significant OR of 1.67 for health worker training (95% CI: 1.38-2.03, p<0.001) underscores the crucial role that education and training play in enhancing surveillance and treatment practices, particularly in combating misinformation and vaccine hesitancy.

When evaluating Pakistan's chances of controlling and eliminating pediatric polio, several crucial tactics become apparent. Improving vaccination efforts, via focused advertising, and tackling vaccine reluctance are critical. Furthermore, encouraging community involvement and mobilization initiatives might improve vaccination uptake and surveillance efficacy. Improving monitoring systems and providing more access to in-depth rehabilitation treatments are essential for reducing long-term effects and achieving better results. Additionally, the discovery of vaccines and innovative approaches to disease management may hasten the elimination of polio. Through the coordinated use of these techniques, Pakistan may get closer to its objective of providing its children with a future free from polio.

This study has a few limitations, primarily its small sample size of 26 patients, which may not fully represent the diversity of pediatric polio cases nationwide, impacting the comprehensive understanding of disease dynamics and surveillance efficacy. Moreover, reliance on retrospective data collection methods such as medical record reviews and interviews could have introduced recall bias and incomplete information, thereby influencing the study's findings and recommendations for public health interventions.

## Conclusions

The demographic, clinical, and epidemiological features of pediatric polio in Pakistan are comprehensively analyzed in this cohort research, which also provides important information on vaccination coverage, illness prevalence, and the efficacy of monitoring. The results highlight the ongoing difficulties in eliminating polio, such as vaccine coverage gaps, a range of clinical presentations, and the disease's long-lasting motor deficits. The research does, however, also shed light on some encouraging opportunities for polio control and eradication, such as bolstering vaccination campaigns, upgrading monitoring systems, and increasing access to rehabilitation treatments. Through the integration of these tactics with focused interventions aimed at mitigating vaccination hesitancy and improving community involvement, Pakistan can achieve the objective of eliminating polio.
